# Improving statistical inference on pathogen densities estimated by quantitative molecular methods: malaria gametocytaemia as a case study

**DOI:** 10.1186/s12859-014-0402-2

**Published:** 2015-01-16

**Authors:** Martin Walker, María-Gloria Basáñez, André Lin Ouédraogo, Cornelus Hermsen, Teun Bousema, Thomas S Churcher

**Affiliations:** 1 Department of Infectious Disease Epidemiology, School of Public Health, Faculty of Medicine (St Mary’s campus), Imperial College London, Norfolk Place, London, W2 1PG UK; 2 Centre National de Recherche et de Formation sur le Paludisme, BP 2208, Ouagadougou, 01 Burkina Faso; 3 Department of Medical Microbiology, Radboud University Medical Centre, Nijmegen, PO Box 9101, 6500 HB Nijmegen, Netherlands; 4 Department of Immunology and Infection, London School of Hygiene and Tropical Medicine, London, UK; 5 MRC Centre for Outbreak Analysis and Modelling, Department of Infectious Disease Epidemiology, School of Public Health, Faculty of Medicine (St Mary’s campus), Imperial College London, Norfolk Place, London, W2 1PG UK

## Abstract

**Background:**

Quantitative molecular methods (QMMs) such as quantitative real-time polymerase chain reaction (q-PCR), reverse-transcriptase PCR (qRT-PCR) and quantitative nucleic acid sequence-based amplification (QT-NASBA) are increasingly used to estimate pathogen density in a variety of clinical and epidemiological contexts. These methods are often classified as semi-quantitative, yet estimates of reliability or sensitivity are seldom reported. Here, a statistical framework is developed for assessing the reliability (uncertainty) of pathogen densities estimated using QMMs and the associated diagnostic sensitivity. The method is illustrated with quantification of *Plasmodium falciparum* gametocytaemia by QT-NASBA.

**Results:**

The reliability of pathogen (e.g. gametocyte) densities, and the accompanying diagnostic sensitivity, estimated by two contrasting statistical calibration techniques, are compared; a traditional method and a mixed model Bayesian approach. The latter accounts for statistical dependence of QMM assays run under identical laboratory protocols and permits structural modelling of experimental measurements, allowing precision to vary with pathogen density. Traditional calibration cannot account for inter-assay variability arising from imperfect QMMs and generates estimates of pathogen density that have poor reliability, are variable among assays and inaccurately reflect diagnostic sensitivity. The Bayesian mixed model approach assimilates information from replica QMM assays, improving reliability and inter-assay homogeneity, providing an accurate appraisal of quantitative and diagnostic performance.

**Conclusions:**

Bayesian mixed model statistical calibration supersedes traditional techniques in the context of QMM-derived estimates of pathogen density, offering the potential to improve substantially the depth and quality of clinical and epidemiological inference for a wide variety of pathogens.

**Electronic supplementary material:**

The online version of this article (doi:10.1186/s12859-014-0402-2) contains supplementary material, which is available to authorized users.

## Background

The development of quantitative molecular methods (QMMs) has allowed the detection and quantification of pathogens at concentrations below the threshold of detection by conventional diagnostic tools [1]. Molecular tools such as quantitative real-time polymerase chain reaction (q-PCR), reverse-transcriptase PCR (qRT-PCR) and quantitative nucleic acid sequence-based amplification (QT-NASBA) are routinely used to estimate the density of a variety of pathogens, including human immunodeficiency virus (HIV), influenza viruses and *Plasmodium* species protozoa which cause malaria. Pathogen density estimates are increasingly being used in epidemiological assessments (for example, to determine viral [2,3] and bacterial [4] transmissibility), clinical management (such as in HIV [5] and bacterial pneumonia [6]), and to assess the effectiveness of control interventions [7,8]. Therefore, it is critically important that the quantitative and diagnostic performance of QMMs is accurately appraised and that point-estimates of pathogen density are accompanied by robust estimates of reliability (uncertainty).

The principles underlying QMMs such as qPCR, qRT-PCR and QT-NASBA are broadly the same. Nucleic acid in a sample is amplified together with a fluorescent probe and the time taken for the reaction to achieve a certain degree of fluorescence—the experimental measurement—is used to estimate the initial quantity of nucleic acid. ‘Absolute’ quantification [9] uses calibration or ‘standard’ curves of test samples with concentrations measured precisely enough to be considered known, so-called calibrators. Typically, this is achieved by diluting a sample of high concentration measured by the available gold standard quantitative diagnostic to yield a ‘dynamic range’ of calibrators typically in the order of 4 to 8 logarithms, a procedure called serial dilution. The alternative ‘relative’ quantification uses an internal reference gene and calculates the relative expression ratio [10]. Based on the theory of nucleic acid amplification, the quantity of nucleic acid in the amplification phase increases exponentially and so plotting the experimental measurement against the logarithm of the calibrators yields a linear relationship. The fitted regression line describing this relationship is called a calibration or standard ‘curve’. Statistical calibration [11] refers to the process of using a calibration curve to estimate an unknown (logarithm of) quantity of interest (here pathogen density) from an experimental measurement.

Quantitative molecular methods have been described as either quantitative or semi-quantitative [12]. In reality, their performance ranges from quantitative and highly accurate, to predominantly qualitative indicators of presence or absence. A cascade of numerous potential sources of uncertainty in laboratory protocol [13] mean that most QMMs lie between these extremes, having intermediate quantitative resolution [14,15]. Regardless of the source, uncertainties manifest in calibration curves with non-negligible (intra-assay) residual error in experimental measurements, and potential inter-assay variability among slopes and intercepts, even when undertaken using standardized protocols within the same laboratory [13]. These errors are widely acknowledged, defined in the MIQE guidelines (minimum information for publication of quantitative real-time PCR experiments) as ‘repeatability’ (intra-assay variance) and ‘reproducibility’ (inter-assay variance) respectively [16], and are broadly indicative of the quantitative and diagnostic performance of the QMM in question.

Despite this, there is a lack of statistical understanding on how exactly such (intra- and inter-assay) errors translate into the reliability of estimated pathogen densities or nucleic acid copy numbers, and into the diagnostic sensitivity (sometimes termed ‘clinical sensitivity’ to distinguish it from ‘analytical sensitivity’ which refers to the minimum number of detectable nucleic acid copies [16]) of the QMM. Indeed, calibration techniques developed in the statistical literature [11] have not been adequately applied in the context of QMMs. By contrast, in applied physical science disciplines, particularly in analytical chemistry, where calibration is also widely used, methodological protocols are more firmly embedded within their statistical foundations [17].

In this paper, statistical calibration techniques are applied, as a case study, to 12 calibration curves derived from 12 QT-NASBA assays (1 curve per assay), generated from a single laboratory [18], and used routinely for estimating the density of *Plasmodium* gametocytes present in human blood (gametocytaemia). The QT-NASBA assay uses time to positivity (TTP) in minutes as an indirect measure of pathogen density; the shorter the TTP, the higher the density. Gametocytaemia density determines host infectivity to mosquito vectors and has major epidemiological implications, ranging from quantifying the contribution of different individuals to the reservoir of infection [19], to assessing the effectiveness of transmission-blocking interventions against malaria. Notwithstanding the importance of QT-NASBA to malariologists, the analytical approaches presented here are more broadly applicable to the absolute quantification of a wide range of pathogens by other QMMs. In particular, it is shown how refinements to the traditional calibration approach using random effects and implemented in a Bayesian framework, enable data (calibration curves) from multiple assays to be combined, yielding substantial improvements in accuracy, reliability and consistency of statistical inference on estimated pathogen densities as well as in diagnostic sensitivity.

## Methods

### Ethical clearance

Data were primarily derived from cultured NF54 gametocytes; natural gametocyte isolates were used from a previously published clinical study that received approval from the Ministry of Health of Burkina Faso (2000/3174/MS/SG/DEP).

### The QT-NASBA technique

Full details on the molecular aspects of the QT-NASBA technique are described elsewhere [18]. Briefly, 50-100 μl of blood is collected; the RNA of gametocytes is extracted and then amplified in the presence of a fluorescence probe. The assay measures time to positivity (TTP) in minutes which is the time it takes for the number of target amplicons detected to exceed a defined threshold. In the context of qPCR, TTP is analogous to the quantification cycle (C_q_), threshold cycle (C_T_), crossing point (C_P_), and take-off point (TOP) [16].

### Assays

A QT-NASBA assay is typically run on a 48-well plate. Here, 39 wells contained test samples of unknown gametocyte density; 3 were reserved for negative controls (water), and 6 wells contained samples of known gametocyte density (calibrators) used to calibrate the TTP-gametocyte density relationship. Experimental data from 12 QT-NASBA assays are analysed in this paper to motivate and illustrate the proposed analytical framework.

### Calibrators

Calibrators were prepared using synchronized, purified mature gametocytes derived from an *in vitro* culture of *P. falciparum* [20]. A starting density of 10^6^ gametocytes per ml was estimated using microscopy and used in 6 tenfold dilution series (10^6^ to 10^1^ gametocytes per ml). Hence, 6 calibrators were included in each of the 12 QT-NASBA assays.

### Calibration curves

**Figure 1:**
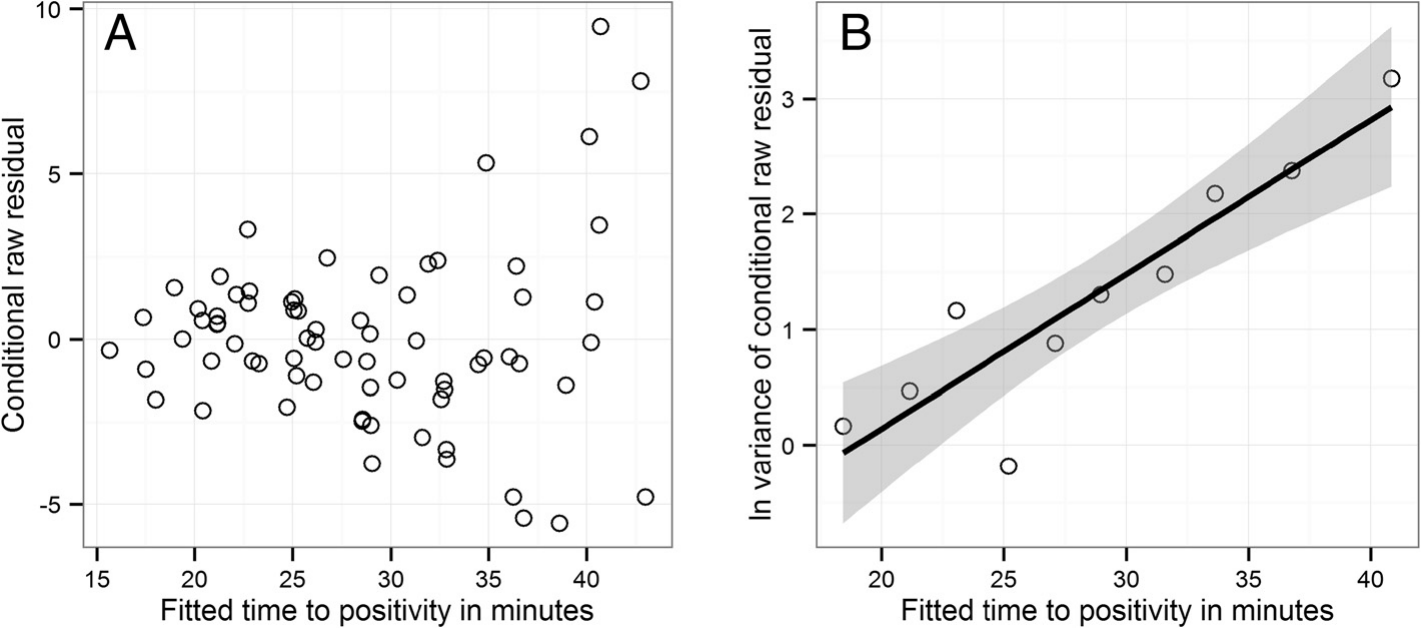
**Heteroscedasticity of calibration or standard curves from quantitative nucleic acid sequence-based amplification (QT-NASBA) assays.** Panel **A** depicts the conditional raw residuals of a homoscedastic linear mixed model with a correlated random intercept and slope (the homoscedastic homologue of the selected Model 7 in Table 2) plotted against the fitted time to positivity (TTP). The data points in panel **B** depict the natural logarithm (ln) of the variance of the residuals presented in panel **A** grouped by deciles (10-quantiles) of fitted TTP. The solid line and shaded area are for presentation purposes only, representing a linear regression ± 2 standard deviations respectively.

The log_10_ (base 10)-transformed density of calibrator *i* = 1,2,…,*n* (here *n* = 6, i.e. 6 calibrators per assay) from assay *j* = 1,2,…,*r* (here *r* = 12, i.e. 12 assays in total), is denoted *x*_*ij*_. The TTP value associated with each calibrator (viz. each *x*_*ij*_) is estimated using QT-NASBA and denoted by the random variable *Y*_*ij*_. The relationship between TTP and pathogen density is described by a linear mixed model (LMM) [21,22] of the form

1 $$ {Y}_{ij}={\beta}_{0j}+{\beta}_{1j}\times \left({x}_{ij}-\overline{x}\right)+{\varepsilon}_{ij}, $$

where $\overline{x}$ is the mean of the calibrators, *ε*_*ij*_ is a normally distributed residual error term with mean 0, and *β*_0*j*_ and *β*_1*j*_ are random effects which follow a multivariate normal distribution with a mean vector of fixed effects **β** = (*β*_0_, *β*_1_), standard deviation (SD) vector **τ** = (*τ*_0_, *τ*_1_) and correlation *ρ*. Setting **τ** = (0, 0), the LMM becomes a linear model (LM) with fixed effects *β*_0*j*_ = *β*_0_ and *β*_1*j*_ = *β*_1_ (no random variation among assay-specific regression coefficients). Homoscedastic (constant) intra-assay residual variance is defined by var(*ε*_*ij*_) = σ^2^. Alternatively, heteroscedasticity (non-constant intra-assay variance) is captured by defining var(*ε*_*ij*_) as a non-constant function. Specifically, and based on inspecting residuals of a fitted model (Figure 1), var(*ε*_*ij*_) is defined as a log-linear function of the mean of ${Y}_{ij},{\mu}_{ij}={\beta}_{0j}+{\beta}_{1j}\times \left({x}_{ij}-\overline{x}\right),$

2 $$ \mathrm{v}\mathrm{a}\mathrm{r}\left({\varepsilon}_{ij}\right)={\upsigma}^2 \exp \left(\gamma {\mu}_{ij}\right), $$

noting that *γ* = 0 reproduces intra-assay homoscedasticity.

### Classical calibration

Statistical calibration [11] concerns making inference on an unknown value of the independent variable (gametocyte density in assay *j*), denoted *X*_0*j*_, from a single experimental observation (a TTP), *y*_0*j*_, or more generally from the mean of *m* observations (TTPs), ${\overline{y}}_{0j}={\Sigma}_i{y}_{0ij}/m$. Solving (1) for *x*_*ij*_ and substituting *x*_*ij*_ and *Y*_*ij*_ for *X*_0*j*_ and ${\overline{y}}_{0j}$ respectively yields

3 $$ {X}_{0j}=\overline{x}+\left({\overline{y}}_{0j}-{\beta}_{0j}-{\varepsilon}_{ij}\right)/{\beta}_{1j}. $$

A point-estimate of *X*_0*j*_, denoted ${\widehat{x}}_{0j},$ is given by replacing *β*_0*j*_ and *β*_1*j*_ in (3) with their so-called empirical best linear unbiased predictors (EBLUPs) [21], denoted *b*_0*j*_ and *b*_1*j*_,

4 $$ {\widehat{x}}_{0j}=\overline{x}+\left({\overline{y}}_{0j}-{b}_{0j}\right)/{b}_{1j}. $$

For the fixed effects LM where **τ** = (0,0), *b*_0*j*_ = *b*_0_ and *b*_1*j*_ = *b*_1_, (4) becomes Eisenhart’s classical calibration estimator and the estimates *b*_0_ and *b*_1_ can be obtained by ordinary least squares.

The classical calibration estimator [23], derived from a linear regression model with normally distributed errors, is the fixed effects version of (4),

5 $$ {\widehat{x}}_0=\overline{x}+\left({\overline{y}}_0-{b}_0\right)/{b}_1. $$

Uncertainty in the estimates *b*_0_ and *b*_1_ propagates into the sampling distribution of ${\widehat{x}}_0$ resulting in a ratio distribution with undefined moments [24,25]. Specifically, if the residual error variance *σ* ^2^ of the calibration curve is known, ${\widehat{x}}_0$ is distributed as the ratio of two normally distributed random variables. More commonly, *σ* ^2^ is estimated as *s* ^2^, and ${\widehat{x}}_0$ is distributed as the ratio of *t* distributed random variables [26]. In practice, the distributional nuances of ${\widehat{x}}_0$ are seldom relevant for ‘high quality’ calibration curves where the absolute magnitude of *β*_1_ is large relative to *σ* ^2^_,_ and there are numerous and adequately dispersed calibrators (i.e. when ${\Sigma}_i{\left({x}_i-\overline{x}\right)}^2$ is large). In such circumstances, *b*_1_ is strongly significantly different from 0. Indeed, conditioning on this event has proved a popular approach to ensure the existence of finite moments of ${\widehat{x}}_0$ [27,28]. By this method, the approximate variance of ${\widehat{x}}_0$ is

6 $$ \mathrm{v}\mathrm{a}\mathrm{r}\left({\widehat{x}}_0\right)\simeq \frac{\sigma^2}{\beta_1^2}\left(\frac{1}{n}+\frac{1}{m}+\frac{{\left({\overline{y}}_0-{\beta}_0\right)}^2}{\beta_1^2{\displaystyle {\sum}_i{\left({x}_i-\overline{x}\right)}^2}}+\frac{3{\sigma}^2}{m{\beta}_1^2{\displaystyle {\sum}_i{\left({x}_i-\overline{x}\right)}^2}}\right), $$

which is used widely, particularly in analytical chemistry, to construct confidence intervals (CIs); usually invoking a *t*-distribution, replacing, respectively, *σ* ^2^ and *β*_1_ with *s* ^2^ and *b*_1_, and assuming that the final term in the parentheses is negligible [17,29,30]. The so-called ‘fiducial’ CI [31] is also popular, derived by finding values of ${\widehat{x}}_0$ that satisfy the bounds of the prediction interval of ${\overline{y}}_0$ at ${\widehat{x}}_0$ [32],

7 $$ \left\{{\widehat{x}}_{0,L},{\widehat{x}}_{0,U}\right\}=\overline{x}+\frac{{\overline{y}}_0-{b}_0}{b_1\left(1-g\right)}\pm \frac{t}{b_1\left(1-g\right)}\sqrt{\left(1-g\right){s}^2\left(\frac{1}{m}+\frac{1}{n}\right)+\frac{s^2{\left({\overline{y}}_0-{b}_0\right)}^2}{b_1{\displaystyle {\sum}_i{\left({x}_i-\overline{x}\right)}^2}}}, $$

where

8 $$ g={t}^2{s}^2/\left({b}_1^2{\displaystyle {\sum}_i{\left({x}_i-\overline{x}\right)}^2}\right), $$

and *t* is the critical value of Student’s *t*-distribution with *n* + *m* −3 degrees of freedom. Term *g* (the ‘*g* statistic’) is important because it inversely measures the ‘performance’ of a calibration curve, assimilating the gradient, the residual variability, and the number and spread of the calibrators into a single metric; as *g* → 0 the variance of ${\widehat{x}}_0$ decreases [33-35]. It is also noteworthy that as *g* → 0, the fiducial CI tends to the CI constructed using the variance approximation. Indeed, the later approach is deemed valid only for *g* < 0.05 [17]. The fiducial limit, although generally more versatile than the variance approximation approach, performs increasingly poorly for increasing *g* [26,36].

### Bayesian calibration

In general, the undefined moments of the ratio distribution *b*_0*j*_ / *b*_1*j*_ create problems for the quantification of SE by traditional (frequentist) approaches, albeit point estimates are largely invariant. This is particularly the case for a LMM where prerequisite estimation of uncertainty in the EBLUPs (*b*_0*j*_ and *b*_1*j*_) is problematic [21]. The Bayesian solution [11] rests on evaluating the posterior distribution of *X*_0*j*_, given by Bayes’ theorem,

9 $$ p\left({X}_{0j}\Big|{\overline{y}}_{0j},\boldsymbol{\uptheta} \right)\propto p\left({\overline{y}}_{0j}\left|{X}_{0j},\boldsymbol{\uptheta} \left)p\right({X}_{0j}\right|\boldsymbol{\uptheta} \right), $$

where **θ** represents in a generic manner the parameters of the hetero- or homoscedastic LM or LMM. Indicated by (9) is that—in the absence of observed ${\overline{y}}_{0j}$—the posterior distribution (hereafter abbreviated to posterior) of *X*_0*j*_ can be simulated via the posterior predictive distribution [37] of *Y* at ${X}_{0j}={x}_{0j},\ p\left({\overline{y}}_{0j}\Big|{X}_{0j}={x}_{0j},\boldsymbol{\uptheta} \right)$, before applying the rearranged regression (3). Therefore, p(*X*_0*j*_|*ӯ*_0*j*_,**θ**) is rewritten as p(*X*_0*j*_|*x*_0*j*_,**θ**). Simulating from p(*X*_0*j*_|*x*_0*j*_,**θ**) in this way enables the performance of calibration curves (summarized in terms of reliability and diagnostic sensitivity) to be evaluated at chosen hypothetical values of the ‘true’ unknown gametocyte density. In general, it is necessary to evaluate p(*X*_0*j*_|*x*_0*j*_,**θ**) numerically by Markov chain Monte Carlo (MCMC) methods.

Simulations were conducted by MCMC sampling implemented in OpenBUGS (http://www.openbugs.net) [38], the currently maintained and updated version of WinBUGS [39]. To reflect the absence of prior information on the parameter values, vague (uninformative) prior distributions were defined for the **θ**: regression coefficients [fixed effects, including those of the log-linear heteroscedastic function defined by (2)] and random effects were assigned normal priors with mean 0 and variance 1000; variance parameters were assigned inverse-gamma priors with shape and rate parameters equal to 0.001, and the covariance matrix **Σ** of linear mixed models was assigned an inverse Wishart distribution with 2 degrees of freedom [37]. Three Markov chains were initialized for each simulation. Visual inspection of the Markov chains, autocorrelation plots and the Gelman-Rubin statistic [37] were used to assess convergence on the parameter posterior distributions and to check that the conclusions were not sensitive to the choice of starting values. In general, the first 2,500 samples from each chain were discarded as ‘burn-in’ and a further 50,000 samples were used to estimate the marginal posterior distributions.

## Results

### Goodness of fit

**Figure 2:**
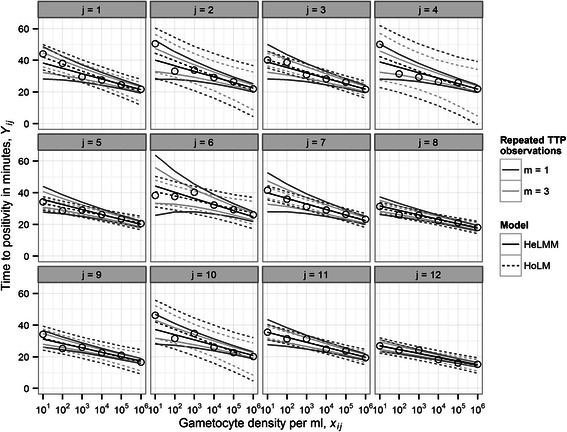
**Calibration or standard curves derived from individual quantitative nucleic acid sequence-based amplification (QT-NASBA) assays.** Panels depict data and fitted calibration curves for assays *j* = 1,2,…,12. Solid and broken lines denote medians and 95% Bayesian credible intervals (BCIs) of the posterior predictive distribution of time to positivity (TTP) calculated from the heteroscedastic linear mixed model (HeLMM) and the homoscedastic linear model (HoLM) respectively (note that for the HoLM these are identical to classical frequentist prediction intervals). Dark and light grey lines correspond to, respectively, BCIs for *m* = 1 TTP observation and the mean of *m* = 3 TTP observations.

Calibration curves fitted to the *Plasmodium* gametocytaemia data from the 12 assays, either individually using a homoscedastic (constant intra-assay variance) linear model (HoLM)—also referred to as the traditional approach—or collectively using a heteroscedastic (dynamic intra-assay variance) linear mixed model (HeLMM), are depicted in Figure 2. Parameter estimates and summary statistics of the HoLMs are given in Table 1. The goodness-of-fit of these models varies considerably among assays, from *R* ^2^ = 97% in assay 7 (assay *j* = 7) to *R* ^2^ = 74% in assay 4 (assay *j =* 4). Reflecting this heterogeneity, the 95% prediction intervals for the mean of *m* TTP observations, ${\overline{y}}_{0j},$ from hypothetical ‘true’ values of log_10_ gametocyte density, *x*_0*j*_, also vary markedly (Figure 2).

**Table 1:** **Summary of the homoscedastic linear model calibration curves fitted by ordinary least squares**

**Curve**	**Intercept** ^**a**^ **,** ***β***_**0**_ **(SE** ^**b**^ **)**	**Slope,** ***β***_**1**_ **(SE)**	**Variance,** ***σ*** ^**2**^	***R*** ^**2**^	***g*** ^**c**^
*j* = 1	30.87 (0.96)	−4.42 (0.56)	5.58	0.94	0.13
*j* = 2	32.46 (1.90)	−4.79 (1.11)	21.75	0.82	0.42
*j* = 3	30.88 (0.64)	−3.80 (0.38)	2.47	0.96	0.08
*j* = 4	30.82 (2.35)	−4.59 (1.37)	33.00	0.74	0.69
*j* = 5	27.00 (0.51)	−2.48 (0.30)	1.54	0.95	0.11
*j* = 6	33.89 (1.16)	−2.69 (0.68)	8.14	0.79	0.50
*j* = 7	31.21 (0.53)	−3.49 (0.31)	1.66	0.97	0.06
*j* = 8	24.09 (0.46)	−2.40 (0.27)	1.25	0.95	0.09
*j* = 9	24.28 (0.91)	−2.98 (0.53)	4.93	0.89	0.24
*j* = 10	30.25 (1.62)	−4.73 (0.95)	15.78	0.86	0.31
*j* = 11	27.71 (0.58)	−3.15 (0.34)	2.00	0.96	0.09
*j* = 12	20.85 (0.58)	−2.51 (0.34)	2.00	0.93	0.14

^a^Calibrators, *x*_*ij*_, were centered about their mean, $\overline{x},$ ensuring that ‘intercept’ terms correspond to the respective estimates at ${x}_{ij}=\overline{x}$.

^b^Standard error.

^c^Calculated using a Student’s critical *t* value at a significance level of 5% and *n* + *m* −3 = 4 + 1–3 degrees of freedom (8).

The HeLMM was selected from several parameterizations of the LMM (including homoscedastic variants) which were compared using the deviance information criterion (DIC) [40] (Table 2). The selected LMM with the lowest DIC included a correlated random intercept and slope, and intra-assay heteroscedastic errors (Model 7 in Table 2). Inclusion of heteroscedasticity yielded particularly large reductions in the DIC of all models compared with their homoscedastic counterparts (Table 2), consistent with an analysis of the residuals from the homoscedastic LMM (Figure 1). Generally, the fitted lines (strictly, posterior means) of the selected HeLMM do not differ substantially from the individually-fitted HoLMs. Posterior means and SDs of estimated parameters of all LMM parameterizations are given in Table 3.

**Table 2:** **Summary of the linear mixed models fitted by Bayesian Markov chain Monte Carlo methods**

**Model**	**Random effects**	**Correlated random effects**	**Heteroscedasticity**	**DIC** ^**a**^
1	Slope	NA^b^	✗	302
2	Intercept	NA	✗	250
3	Intercept; slope	✗	✗	248
4	Intercept; slope	✓	✗	243
5	Intercept	NA	✓	196
6	Intercept; slope	✗	✓	189
7	Intercept; slope	✓	✓	183

Symbols: ✓, included in the model; ✗ not included in the model.

^a^Deviance information criterion.

^b^Not applicable.

**Table 3:** **Parameter estimates from the linear mixed models fitted by Bayesian Markov chain Monte Carlo methods**

**Model**	**Fixed effects**		**Random effects**	**Log-linear variance**	
	**Intercept,** ***β***_**0**_ **(SD** ^**a**^ **)**	**Slope,** ***β***_**1**_ **(SD)**	**Covariance matrix, Σ (SD)**	**exp(intercept),** ***σ*** ^**2**^ **(SD)**	**Slope, γ (SD)**
1	28.7 (0.6)	−3.5 (0.4)	$$ \left[\begin{array}{cc}\hfill 0\hfill & \hfill 0\hfill \\ {}\hfill 0\hfill & \hfill 0.1\kern0.5em (0.3)\hfill \end{array}\right] $$	23.3 (4.1)	0
2	28.7 (1.2)	−3.5 (0.2)	$$ \left[\begin{array}{cc}\hfill 16.8\;(9.9)\hfill & \hfill 0\hfill \\ {}\hfill 0\hfill & \hfill 0\hfill \end{array}\right] $$	10 (1.9)	0
3	28.7 (1.3)	−3.5 (0.3)	$$ \left[\begin{array}{cc}\hfill 17.1\kern0.22em (10.1)\hfill & \hfill 0\hfill \\ {}\hfill 0\hfill & \hfill 0.3\;(0.4)\hfill \end{array}\right] $$	9.3 (1.9)	0
4	28.7 (1.2)	−3.5 (0.3)	$$ \left[\begin{array}{cc}\hfill 14.6\kern0.22em (8.0)\hfill & \hfill -2.5\kern0.5em (1.6)\hfill \\ {}\hfill -2.5\kern0.5em (1.6)\hfill & \hfill 0.5(0.4)\hfill \end{array}\right] $$	9.0 (1.7)	0
5	28.1 (1.0)	−3.0 (0.2)	$$ \left[\begin{array}{cc}\hfill 12.1\kern0.22em (6.8)\hfill & \hfill 0\hfill \\ {}\hfill 0\hfill & \hfill 0\hfill \end{array}\right] $$	0.0 (0.0)	0.2 (0.0)
6	28.2 (1.1)	−3.1 (0.2)	$$ \left[\begin{array}{cc}\hfill 14.0\kern0.22em (8.0)\hfill & \hfill 0\hfill \\ {}\hfill 0\hfill & \hfill 0.2\;(0.2)\hfill \end{array}\right] $$	0.0 (0.0)	0.2 (0.0)
7	28.4 (1.1)	−3.3 (0.2)	$$ \left[\begin{array}{cc}\hfill 14.2\kern0.22em (8.3)\hfill & \hfill -1.4\kern0.5em (1.3)\hfill \\ {}\hfill -1.4\kern0.5em (1.3)\hfill & \hfill 0.2\;(0.2)\hfill \end{array}\right] $$	0.0 (0.0)	0.2 (0.0)

^a^Standard deviation.

### Performance and variability

**Figure 3:**
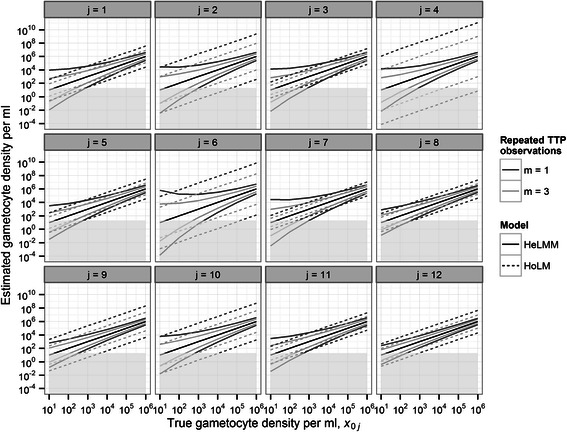
**Reliability of** ***Plasmodium falciparum*** **gametocyte densities estimated by individual quantitative nucleic acid sequence-based amplification (QT-NASBA).** Panels depict hypothetical ‘true’ and estimated gametocyte densities for assays *j* = 1, 2,…, 12. Solid and broken lines denote, respectively, medians and 95% Bayesian credible intervals (BCIs) of the gametocytaemia posterior distributions calculated from the heteroscedastic linear mixed model (HeLMM) and the homoscedastic linear model (HoLM), as defined in the main text. Dark and light grey lines correspond to, respectively, BCIs for *m* = 1 time to positivity (TTP) observation and the mean of *m* = 3 TTP observations. The light-grey shaded area indicates the detection threshold of 1 gametocyte per 0.05 ml of blood.

**Figure 4:**
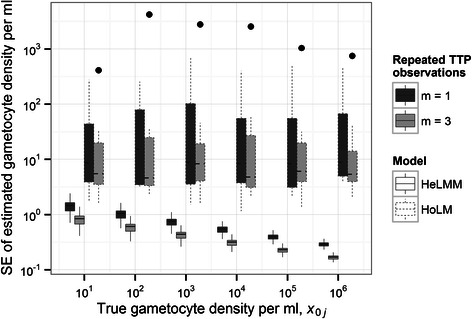
**Variability of** ***Plasmodium falciparum*** **gametocyte density reliability estimates from quantitative nucleic acid sequence-based amplification (QT-NASBA).** The boxes surrounded by dashed lines and solid lines depict, respectively, the distribution of assay-specific gametocyte density posterior standard deviations (analogous to, and labelled as, frequentist standard errors, SEs) derived from the 12 individually-fitted homoscedastic linear models (HoLMs) and the heteroscedastic linear mixed model (HeLMM). Boxes span from the 25^th^ to the 75^th^ percentiles (the interquartile range) of the estimated SEs and whiskers a further 1.5 × the interquartile range. Points outside of this range are indicated and horizontal bars (broken and solid) denote the medians. Boxes shaded dark grey and light grey correspond to, respectively, estimates derived from a single time to positivity (TTP) observation (*m* = 1) or the mean of 3 TTP observations (*m* = 3).

The performance of calibration curves is summarized in terms of reliability and diagnostic sensitivity. Specifically, inference is based on the numerical (MCMC) approximation of the gametocyte density posterior conditioned on known *x*_0*j*_ and parameters **θ**, p(*X*_0*j*_|*x*_0*j*_,**θ**). Reliability is measured by either the 95% Bayesian credible interval (BCI) (Figure 3) or the SD (hereafter referred to in a frequentist manner as a standard error, SE) (Figure 4) of p(*X*_0*j*_|*x*_0*j*_,**θ**). Diagnostic sensitivity is quantified by the percentage of realizations from p(*X*_0*j*_|*x*_0*j*_,**θ**) greater than 1 gametocyte per 0.05 ml volume of test sample (the volume in a single well of a plate used to run each assay) (Figure 3).

**Figure 5:**
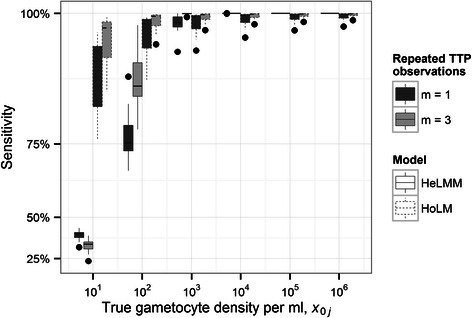
**Sensitivity and consistency of detecting** ***Plasmodium falciparum*** **gametocytes by quantitative nucleic acid sequence-based amplification (QT-NASBA).** The boxes surrounded by dashed lines depict, respectively, the distribution of assay-specific sensitivities derived from the 12 individually-fitted homoscedastic liner models (HoLMs) and the heteroscedastic linear mixed model (HeLMM). Boxes span from the 25^th^ to the 75^th^ percentiles (the interquartile range) of the estimated sensitivities and whiskers a further 1.5 × the interquartile range. Points outside of this range are indicated and horizontal bars (broken and solid) indicate medians. Boxes shaded dark grey and light grey correspond to, respectively, estimates derived from a single time to positivity (TTP) observation (*m* = 1) or the mean of 3 TTP observations (*m* = 3).

Estimates of reliability (Figure 3; Figure 4) and sensitivity (Figure 5) derived from HoLMs are highly heterogeneous, reflecting substantial variation in performance among calibration curves (assays). This heterogeneity is also captured by the so-called *g* statistic (8) (Table 1) which expresses the performance of each HoLM in a single metric. The *g* statistic also indicates when uncertainty intervals constructed using classical frequentist approximations [e.g. Equation (7)] will prove satisfactory (Additional file 1). By contrast, calibration using the selected HeLMM (Model 7, Table 2) yields less variable estimates of reliability (Figure 3; Figure 4) and sensitivity (Figure 5) by the (random effects) assumption that data from different assays are statistically related; that is, calibration curves are realizations from an underlying distribution, each with a different intercept and slope (Table 2), but with the same (heteroscedastic) intra-assay variance function (2).

Intra-assay heteroscedasticity decreases the SE of the gametocyte density (decreases variance, Figure 1) with increasing *x*_0*j*_ in a density-dependent manner (Figure 3; Figure 4). Consequently, the reliability of the HeLMM-derived estimates at low *x*_0*j*_, while quite homogeneous among assays, is only moderately superior to the majority of HoLM-derived estimates. Conversely, at high *x*_0*j*_, HeLMM-derived estimates are markedly more reliable (Figure 3, Figure 4). Heteroscedasticity also introduces density dependence into diagnostic sensitivity, reducing the sensitivity estimated from the HeLMM compared to the HoLM at low *x*_0*j*_, and *vice versa*. Indeed, at 10 gametocytes per ml, the HeLMM-derived estimates of sensitivity are between 25% and 50% compared to the HoLM-derived estimates which are all greater than 75% (Figure 4). Unsurprisingly, the SE and sensitivity estimated by both models over the range of *x*_0*j*_ is improved by increasing *m* replica TTP observations (Figure 5).

## Discussion

The availability of QMMs has led to a new dimension in clinical and epidemiological research, in which pathogen densities can be detected and quantified below the thresholds of conventional diagnostics. Despite broad awareness of the numerous potential sources of uncertainty inherent to QMMs, even implemented using standardized experimental protocols, there has hitherto been a lack of clear explanation on how these uncertainties: (a) manifest as intra- and inter-assay errors in calibration curves; (b) project onto the reliability (uncertainty) of estimated pathogen densities and the diagnostic sensitivity of the QMM, and (c) should be handled statistically to estimate robust measures of quantitative and diagnostic performance. These gaps perhaps explain why estimates of reliability and sensitivity—essential metrics for statistical inference—seldom accompany point pathogen density estimates. The presented analysis serves to address these issues by calling attention to calibration methods developed in the statistical literature and refining these techniques to develop a novel and powerful modelling framework.

### Calibration curves, reliability and sensitivity

Previous studies on the reliability of QMM-derived estimates have focused predominantly on uncertainty and intra-assay variability in nucleic acid amplification efficiency [41-43], rather than uncertainty in measures of estimated nucleic acid concentrations or pathogen densities. This is understandable given the uppermost importance of a consistent amplification efficacy for analytical approaches designed for relative quantification [10] and because, for microparasitic pathogens (viruses, bacteria, protozoa), relative quantification, or just detection (presence or absence), is often considered sufficient in clinical contexts [42]. However, in terms of absolute quantification, variation in amplification efficiency—which manifests as inter-assay variability among the slopes of calibration curves—does not determine the reliability of estimates *per se*. This assertion is embodied by the *g* statistic, a single-metric indicator of the ‘performance’ of a simple linear calibration curve (a HoLM) which influences in a non-linear manner the reliability of derived estimates and includes all the components of a desirable, ‘high performing’ calibration curve; one with a steep slope, numerous calibrators spread over a wide dynamic range, and a small degree of residual variability of experimental measurements.

The diagnostic sensitivity of a QMM is inextricably associated with the reliability of estimated pathogen densities. That is, purely by chance, the number of pathogens in a positive sample may be estimated, via a calibration curve, as less than 1. The probability of this occurrence defines diagnostic sensitivity. The results presented here indicate that the uncertainty in QT-NASBA point-estimates increases with decreasing gametocyte densities in a density-dependent manner. The compounding of (initially small) errors throughout the dilution series is a likely and possibly predominant reason for this; at each dilution, additional uncertainty is introduced in the number of ‘known’ gametocytes, error which propagates into the variability of the experimental measurements (TTPs). The net result is that the diagnostic sensitivity of the assay is also density-dependent, decreasing with decreasing gametocyte density (Figure 4). This finding is consistent with other QMMs used to quantify the density of a variety of pathogens [44-46], indicating a general result. It is thus important to emphasize that failing to capture accurately, using a statistical model, systematic changes in the residual variation of experimental measures about a calibration line (intra-assay variance) (Figure 1) risks inaccurately estimating reliability (Figure 4) and diagnostic sensitivity (Figure 5).

### Statistical dependence of calibration curves

Heterogeneity in the quality of calibration curves derived from different QMM assays is inescapable; consequently, variation in estimates of reliability and sensitivity (collectively referred to as ‘performance’) is also inevitable. This is the case in state-of-the-art laboratories using rigorous and standardized experimental protocols [13], and even more so in difficult field settings [47]. Refinements to experimental protocols, including statistically-driven efforts to optimize the number and distribution of calibrators [34], will reduce but not eliminate variation in the quality of calibration curves. Furthermore, the impact of quality on performance is intensified by the practice of using relatively few calibrators per assay (here 6 calibrators were used per 48-well plate, 6 to 8 being commonplace).

The ‘traditional’ calibration approach, whereby calibration curves from different assays are treated as statistically independent, yields considerable variation in estimates of performance. This is compounded with the ubiquitous assumption of homoscedasticity (constant intra-assay variance) of experimental measures—despite observations that SEs tend to be greater at low nucleic acid concentrations or densities (density dependence) of a variety of pathogens [41,44,48]—resulting in performance estimates that are inaccurate as well as heterogeneous. This presents particular difficulties to studies concerned with inference at low pathogen densities [19,49], not only because this is where the problem is worst, but also because this is precisely where QMMs are preferred for their superior detection sensitivity compared with conventional diagnostics.

The problem of variable performance estimates is resolved using mixed model techniques [21,22] adapted to statistical calibration [50]. Mixed models treat calibration curves from different assays as statically related, permitting inference on simultaneous analysis of all data derived from a set of assays run at a particular time or in a particular laboratory. (In principle, the effect of laboratory, time and any other measurable covariate could be incorporated into the framework presented here, although this is beyond the scope of the current paper.) The problem of inaccurate performance estimates is resolved by departing from the assumption of constant intra-assay variability and explicitly modelling density dependence in the residual variance of experimental measures about calibration curves (Figure 1), a construct that can be embedded within the mixed model framework. The resulting heteroscesdastic mixed model comes, of course, with the requirement to justify the modelling assumptions and is more technically challenging and time consuming to implement than a traditional calibration approach, which is based on the simple linear regression model. Nevertheless, the Bayesian MCMC techniques that provide the versatility to implement almost arbitrarily complex custom-built mixed models are now readily accessible in statistical software packages such as WinBUGS [39], OpenBUGS [38], and JAGS [51]. In the end the decision about whether or not to conduct the more time consuming Bayesian analysis will depend on the *g* statistic (a value which should always be quoted) and the degree of precision required from the assay.

### The hazards of ‘quality control’

Hitherto, the issue of variable quality calibration curves and the resulting heterogeneous assay performance has been addressed under the broad banner of ‘quality control’. Strategies have included *post hoc* exclusion of ‘outlying’ calibration data points [41,44] and vetting of calibration curves, as practiced for quantification of hepatitis B and Epstein-Barr viraemias [34]. Both approaches are statistically equivocal unless there are measured independent variables (covariates) to explain the ‘unexpected’ data. For example, exclusion of the poorest quality calibration curve from assay 4 (see Figure 2 and Table 1) would have: (a) made valid statistical inference on the value of an unknown gametocyte density run on that particular assay extremely difficult, but more importantly (b) rendered the estimated performance of all other assays—which are informed by the quality of all the calibration curves—overly optimistic. In essence, the calibration data from assay 4 were observed without *a priori* information to explain their somewhat ‘unexpected’ or ‘outlying’ nature and so they should contribute information to the estimated performance of replica assays like any other. Myriad explanatory variables such as time, reagent batch, technician etc. could be legitimately included in the analysis and may demonstrate that quality, and hence performance, is genuinely better or worse under particular circumstances.

## Conclusions

Taking the quantification of gametocytaemia by QT-NASBA in *P. falciparum* infections as an example, this paper illustrates that: (a) the reliability of estimated pathogen densities, and the diagnostic sensitivity of a QMM, which together define performance, depend on properties of assay-specific calibration curves, namely the slope, the number and spread of calibrators, and the residual variability of experimental measurements; (b) performance is density-dependent if intra-assay residual variability is dynamic over the range of the calibrators, and if density dependence is ignored, estimates of performance will be inaccurate; (c) random variation in the quality of calibration curves from different QMM assays produces variable performance estimates, hampering robust statistical inference; (d) the relatedness of calibration curves derived from replica assays can be exploited, using mixed models, to improve the reliability and consistency of results. Together, these insights demonstrate that investing in appropriate and powerful statistical techniques, ideally as part of routine analysis, can greatly facilitate the interpretation of molecularly-derived estimates of pathogen density, ultimately improving inference in a wide range of clinical and epidemiological contexts.

## Additional file

Additional file 1:
**Comparing uncertainty intervals of** ***Plasmodium falciparum*** **gametocyte densities estimated using a classical frequentist technique or a Bayesian Markov chain Monte Carlo approach.**

## References

[1] Tang YW, Procop GW, Persing DH (1997). Molecular diagnostics of infectious diseases. Clin Chem.

[2] Ferguson NM, Cummings DA, Cauchemez S, Fraser C, Riley S, Meeyai A, Iamsirithaworn S, Burke DS (2005). Strategies for containing an emerging influenza pandemic in Southeast Asia. Nature.

[3] Fraser C, Hollingsworth TD, Chapman R, de Wolf F, Hanage WP (2007). Variation in HIV-1 set-point viral load: epidemiological analysis and an evolutionary hypothesis. Proc Natl Acad Sci U S A.

[4] Wain J, Diep TS, Ho VA, Walsh AM, Nguyen TT, Parry CM, White NJ (1998). Quantitation of bacteria in blood of typhoid fever patients and relationship between counts and clinical features, transmissibility, and antibiotic resistance. J Clin Microbiol.

[5] World Health Organization (2013). Consolidated guidelines on the use of antiretroviral drugs for treating and preventing HIV infection: recommendations for a public health approach. Book Consolidated guidelines on the use of antiretroviral drugs for treating and preventing HIV infection: recommendations for a public health approach.

[6] Woodhead M, Blasi F, Ewig S, Garau J, Huchon G, Ieven M, Ortqvist A, Schaberg T, Torres A, van der Heijden G, Read R, Verheij TJ, Joint Taskforce of the European Respiratory Society and European Society for Clinical Microbiology and Diseases Infectious (2011). Guidelines for the management of adult lower respiratory tract infections–full version. Clin Microbiol Infect.

[7] Murray JS, Elashoff MR, Iacono-Connors LC, Cvetkovich TA, Struble KA (1999). The use of plasma HIV RNA as a study endpoint in efficacy trials of antiretroviral drugs. AIDS.

[8] Mens PF, Sawa P, van Amsterdam SM, Versteeg I, Omar SA, Schallig HD, Kager PA (2008). A randomized trial to monitor the efficacy and effectiveness by QT-NASBA of artemether-lumefantrine versus dihydroartemisinin-piperaquine for treatment and transmission control of uncomplicated *Plasmodium falciparum* malaria in western Kenya. Malar J.

[9] Bustin SA (2000). Absolute quantification of mRNA using real-time reverse transcription polymerase chain reaction assays. J Mol Endocrinol.

[10] Schmittgen TD, Livak KJ (2008). Analyzing real-time PCR data by the comparative C(T) method. Nat Protoc.

[11] Osborne C (1991). Statistical calibration: a review. Int Stat Rev.

[12] Ferre F (1992). Quantitative or semi-quantitative PCR: reality versus myth. PCR Methods Appl.

[13] Nolan T, Hands RE, Bustin SA (2006). Quantification of mRNA using real-time RT-PCR. Nat Protoc.

[14] Khairnar K, Martin D, Lau R, Ralevski F, Pillai DR (2009). Multiplex real-time quantitative PCR, microscopy and rapid diagnostic immuno-chromatographic tests for the detection of *Plasmodium spp*: performance, limit of detection analysis and quality assurance. Malar J.

[15] El Mubarak HS, De Swart RL, Osterhaus AD, Schutten M (2005). Development of a semi-quantitative real-time RT-PCR for the detection of measles virus. J Clin Virol.

[16] Bustin SA, Benes V, Garson JA, Hellemans J, Huggett J, Kubista M, Mueller R, Nolan T, Pfaffl MW, Shipley GL, Vandesompele J, Wittwer CT (2009). The MIQE guidelines: minimum information for publication of quantitative real-time PCR experiments. Clin Chem.

[17] Miller JN (1991). Basic statistical methods for analytical chemistry part 2. Calibration and regression methods* A review. Analyst.

[18] Schneider P, Schoone G, Schallig H, Verhage D, Telgt D, Eling W, Sauerwein R (2004). Quantification of *Plasmodium falciparum* gametocytes in differential stages of development by quantitative nucleic acid sequence-based amplification. Mol Biochem Parasitol.

[19] Churcher TS, Bousema T, Walker M, Drakeley C, Schneider P, Ouedraogo AL, Basanez MG (2013). Predicting mosquito infection from *Plasmodium falciparum* gametocyte density and estimating the reservoir of infection. Elife.

[20] Ponnudurai T, Lensen AH, Meis JF, Meuwissen JH (1986). Synchronization of *Plasmodium falciparum* gametocytes using an automated suspension culture system. Parasitology.

[21] Jiang J (2007). Linear and Generalized Linear Mixed Models and Their Appications.

[22] McCulloch CE, Searle SR (2001). Generalized, Linear and Mixed Models.

[23] Eisenhart C (1939). The interpretation of certain regression methods and their use in biological and industrial research. Ann Math Stat.

[24] Nadarajah S (2006). On the ratio *X*/*Y* for some elliptically symmetric distributions. J Multivar Anal.

[25] Marsaglia G (2006). Ratios of normal variables. J Stat Softw.

[26] McCulloch JH (2010). Posterior Confidence Intervals in Linear Calibration Problems: Calibrating the Thompson Ice Core Index.

[27] Naszódi LJ (1978). Elimination of the bias in the course of calibration. Technometrics.

[28] Shulka GK (1972). On the problem of calibration. Technometrics.

[29] Danzer K, Currie LA, Chem CGAA (1998). Guidelines for calibration in analytical chemistry - Part 1. Fundamentals and single component calibration (IUPAC recommendations 1998). Pure Appl Chem.

[30] Massart DL, Vandeginste BGM, Deming SN, Michotte Y, Kaufman L (1988). Chemometrics: a textbook.

[31] Fieller EC (1954). Some problems in interval estimation. J R Stat Soc Series B Stat Methodol.

[32] Graybill FA (1976). Theory and Application of the Linear Model.

[33] De Beer JO, De Beer TR, Goeyens L (2007). Assessment of quality performance parameters for straight line calibration curves related to the spread of the abscissa values around their mean. Anal Chim Acta.

[34] Lai KK, Cook L, Krantz EM, Corey L, Jerome KR (2005). Calibration curves for real-time PCR. Clin Chem.

[35] Vankeerberghen P, Smeyers-Verbeke J (1992). The quality coefficient as a tool in decisions about the quality of calibration in graphite furnace atomic absorption spectrometry. Chemom Intell Lab Syst.

[36] Hoadley B (1970). A Bayesian look at inverse regression. J Am Stat Assoc.

[37] Gelman A, Carlin JB, Stern HS, Rubin DB (2004). Bayesian Data Analysis.

[38] Lunn D, Spiegelhalter D, Thomas A, Best N (2009). The BUGS project: Evolution, critique and future directions. Stat Med.

[39] Lunn DJ, Thomas A, Best NG, Spiegelhalter DJ (2000). WinBUGS - a Bayesian modelling framework: concepts, structure and extensibility. Stat Comput.

[40] Spiegelhalter DJ, Best NG, Carlin BR, van der Linde A (2002). Bayesian measures of model complexity and fit. J Roy Stat Soc B Stat Meth.

[41] Karlen Y, McNair A, Perseguers S, Mazza C, Mermod N (2007). Statistical significance of quantitative PCR. BMC Bioinformatics.

[42] Yuan JS, Reed A, Chen F, Stewart CN (2006). Statistical analysis of real-time PCR data. BMC Bioinformatics.

[43] Ruijter JM, Ramakers C, Hoogaars WM, Karlen Y, Bakker O, van den Hoff MJ, Moorman AF (2009). Amplification efficiency: linking baseline and bias in the analysis of quantitative PCR data. Nucleic Acids Res.

[44] Niesters HG (2001). Quantitation of viral load using real-time amplification techniques. Methods.

[45] Cohen JF, Chalumeau M, Levy C, Bidet P, Thollot F, Wollner A, Bingen E, Cohen R (2012). Spectrum and inoculum size effect of a rapid antigen detection test for group A streptococcus in children with pharyngitis. PloS One.

[46] Wampfler R, Mwingira F, Javati S, Robinson L, Betuela I, Siba P, Beck HP, Mueller I, Felger I (2013). Strategies for Detection of *Plasmodium* species Gametocytes. PLoS One.

[47] Fiscus SA, Cheng B, Crowe SM, Demeter L, Jennings C, Miller V, Respess R, Stevens W (2006). HIV-1 viral load assays for resource-limited settings. PLoS Med.

[48] Schneider P, Wolters L, Schoone G, Schallig H, Sillekens P, Hermsen R, Sauerwein R (2005). Real-time nucleic acid sequence-based amplification is more convenient than real-time PCR for quantification of *Plasmodium falciparum*. J Clin Microbiol.

[49] Okell LC, Bousema T, Griffin JT, Ouedraogo AL, Ghani AC, Drakeley CJ (2012). Factors determining the occurrence of submicroscopic malaria infections and their relevance for control. Nat Commun.

[50] Sivaganesan M, Seifring S, Varma M, Haugland RA, Shanks OC (2008). A Bayesian method for calculating real-time quantitative PCR calibration curves using absolute plasmid DNA standards. BMC Bioinformatics.

[51] Plummer M, Hornik K, Leisch F, Zeileis A (2003). JAGS: A program for analysis of Bayesian graphical models using Gibbs sampling. Proceedings of the 3rd International Workshop on Distributed Statistical Computing; Vienna, Austria.

