# Supplementary material for: Improving statistical inference on pathogen densities estimated by quantitative molecular methods: malaria gametocytaemia as a case study
Source: BMC Bioinformatics. 2015 Jan 16;16:5. doi: 10.1186/s12859-014-0402-2 (PMC4307378; doi:10.1186/s12859-014-0402-2)
Supplement: Additional file 1: — Comparing uncertainty intervals of Plasmodium falciparum gametocyte densities estimated using a classical frequentist technique or a Bayesian Markov chain Monte Carlo approach. [file 12859_2014_402_MOESM1_ESM.docx]

**Improving statistical inference on pathogen densities estimated by quantitative molecular methods: malaria gametocytaemia as a case study**

**Martin Walker, María-Gloria Basáñez, André Lin Ouédraogo, Cornelus Hermsen, Teun Bousema_,_ and Thomas S. Churcher**

# Additional file 1

## Comparing uncertainty intervals of *Plasmodium falciparum* gametocyte densities estimated using a classical frequentist technique or a Bayesian Markov chain Monte Carlo approach


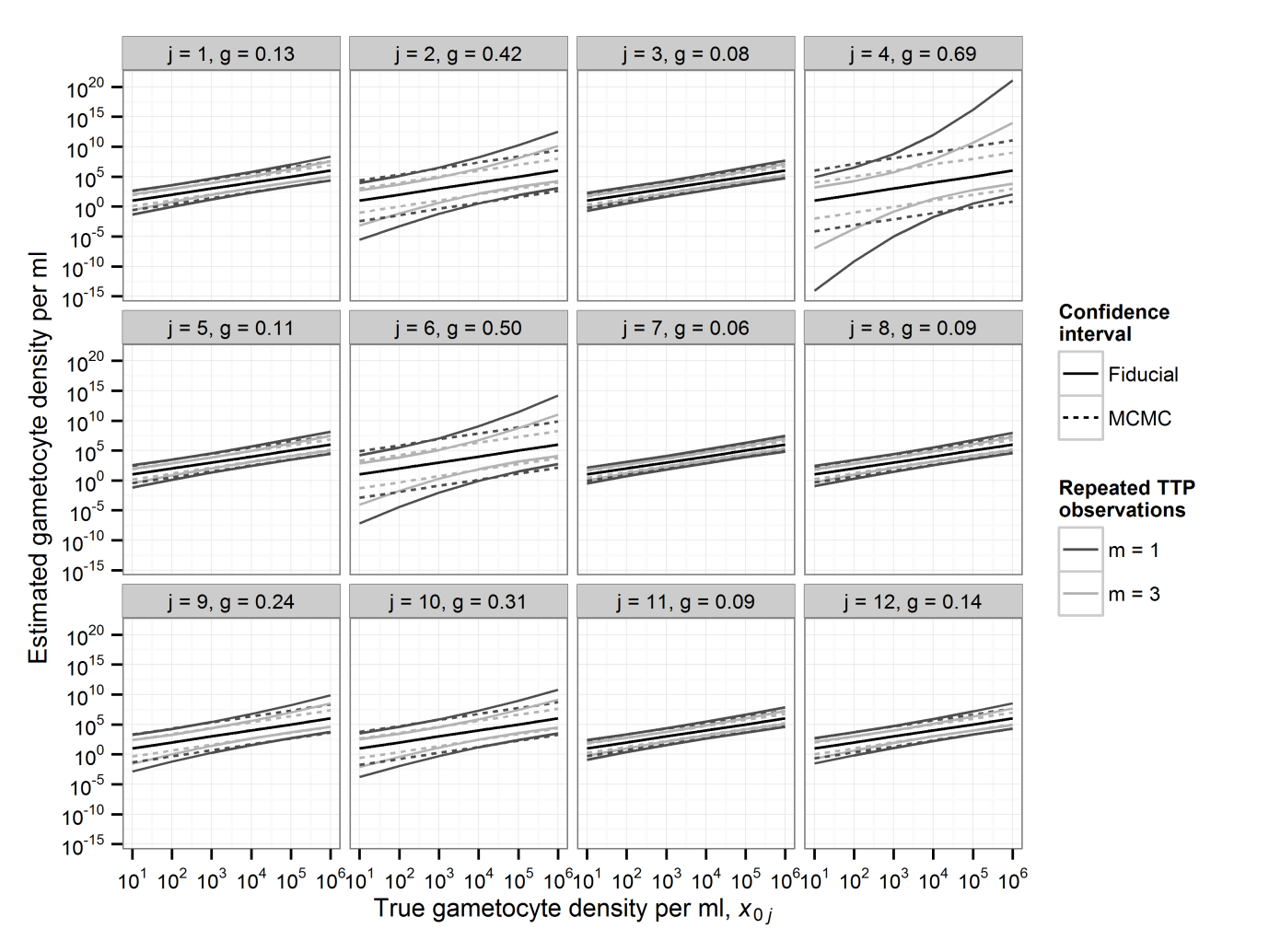


## Figure 1 - Reliability of *Plasmodium falciparum* gametocyte densities estimated by individual quantitative nucleic acid sequence-based amplification (QT-NASBA)

Panels depict hypothetical ‘true’ and estimated gametocyte densities for assays *j* = 1,2,…,12, alongside the *g* statistics (see Table 1 in the main text) for each corresponding calibration curve. Solid lines indicate medians and 95% fiducial prediction intervals [see Equation (7) in the main text] of the estimated gametocytaemia calculated from the homescedastic linear model (HoLM). Dashed lines indicate the medians and 95% Bayesian credible intervals (BCIs) of the corresponding gametocytaemia posterior distributions, again calculated from the HoLM. Dark and light grey lines correspond to, respectively, uncertainty intervals for *m* = 1 time to positivity (TTP) observation and the mean of *m*= 3 TTP observations.
